# Comparison Among Food By-Products to Improve the Shelf Life of a Fresh Burger Based on Shelled Shrimps

**DOI:** 10.3390/foods13213468

**Published:** 2024-10-29

**Authors:** Olimpia Panza, Amalia Conte, Matteo Alessandro Del Nobile

**Affiliations:** 1 Department of Humanistic Studies, Letters, Cultural Heritage, Educational Sciences, University of Foggia, Via Arpi, 71121 Foggia, Italy; olimpia.panza@unifg.it; 2 Department of Economics, Management and Territory, University of Foggia, Via da Zara, 71121 Foggia, Italy; matteo.delnobile@unifg.it

**Keywords:** by-products, shrimp-based burger, shelf life, convenient food, sustainability

## Abstract

Pomegranate peels, fig peels, and by-products from turnip greens were used as novel ingredients in burgers based on shelled shrimps. With the aim, a control without any by-products and three fortified samples with 7.5% (*w*/*w*) by-product were realized. To verify the benefic effects of by-product addition on the chemical quality of burgers, total phenols, total flavonoids, and antioxidant activity were measured in both the control and fortified samples. In addition, during storage under refrigerated conditions, the microbiological proliferation of the main spoilage bacteria, the pH, and the sensory acceptability were properly monitored in all the samples. Results from chemical analyses confirmed that the nutritional level of shrimp-based burgers enriched with by-products was better than that of the control. Burgers with peels from pomegranate recorded the best results (2.67 ± 0.24 mg GAE/g dw, 1.62 ± 0.21 mg QE/g dw, and 12.63 ± 0.41 mg Trolox/g dw for total phenols, flavonoids, and antioxidant activity, respectively). From the microbiological point of view, the bacterial proliferation was always more rapid in the control than in the other samples. Among the by-products, the pomegranate peels better delayed the spoiling phenomena, even though mesophilic and psychrotrophic bacteria grew abundantly and rapidly in all the products, thus reducing the differences among samples. All the burgers maintained sensory acceptability for more than two weeks, regardless of the by-product addition. Considering both microbiological stability and sensory properties, the shelf life of this product was calculated to be around 1 week for the control burger, 8.5 days for both burgers with fig peels and by-products from turnip greens, and 9 days for the shrimp-based burger fortified with pomegranate peels.

## 1. Introduction

Fish and fish products are considered by the scientific community to be among the healthiest foods due to their high nutritional properties. Therefore, their consumption is increasingly widespread, as a guarantee of healthy and balanced diet [1]. Among shellfish fisheries, shelled shrimps are considered rich in proteins and very low in fat. In particular, the shrimp tissues consist of highly unsaturated fatty acids, which are essential in the human diet [2]. However, shelled shrimps are a highly perishable seafood [3,4]. Fresh shrimps are usually prone to changes in microbial, physical, and biochemical quality during storage. The main factors are the high-water content, the free amino acids, and other non-protein nitrogenous compounds [5]. All these factors drastically contribute to reducing fresh shrimps’ shelf life. In addition, melanosis (discoloration) caused by the polymerization of phenols into insoluble black pigments is another important factor causing observable fresh shrimp deterioration [6].

Despite the growing attention toward a healthy diet, the time available to cook is increasingly limited due to an increase in the working women’s population [7]. This aspect confirms the consumer trend towards greater product diversification, such as easily prepared, ready-to-cook, and ready-to-eat fish products. It also justifies the need to develop novel technologies to extend product shelf life without affecting health properties, sensory quality, nutritional standards, and high convenience [8]. In this context, fresh seafood burgers are the most sought-after processed products, being easy to consume through quick cooking and representing an attractive alternative to the consumption of fresh fish [9]. A lot of research has been carried out on fresh fish burgers to preserve and prolong their shelf life, using a proper combination of active preservative and modified atmosphere packaging [10,11,12,13,14,15]. More recently, some efforts have been made to preserve fish products using by-products or their extracts. With this aim, Cedola et al. [16] focused on the development of burgers composed of tuna fillets enriched with industrial olive oil by-products to evaluate microbial and sensory preservation. In another two research studies carried out according to the zero-waste approach, cod burgers were enriched with all parts of pomegranate (peels and arils) or prickly pears (peels and pulp) to extend the shelf life [17,18]. Further studies have been proposed on cod fillets breaded with olive oil by-products [19] and pomegranate by-products [20] to experiment with a new form of preservation through an alternative, active, and natural breading. No experimental research has been carried out on the by-products applied to shrimp-based food as a preservation strategy and more in general, no articles appeared on the shelf life of this type of food. The comparison among different by-products represents another element of novelty for the literature on the topic because the antioxidant and antimicrobial properties of fruit and vegetable residues need to be assessed in real food applications to better highlight their potential under working conditions. Among the articles dealing with shrimp products, one of the first examples is the study of Juemanee et al. [21], who made shrimp-based burgers breaded with hydrocolloids to improve the texture parameters, and the most recent is the research of Suarez et al. [22], who tested the addition of chia seeds in shrimp patties to improve product quality. Shahin et al. [7] proposed the use of shrimps to enhance carp-based meat, carp being an underestimated fish variety, with low nutritional value and undesired sensory properties.

The research aimed to verify the effects of by-product addition to fresh burgers made up of shelled shrimps on both microbiological and sensory evolution, during storage under a refrigerated temperature. The attention was focused on three different by-products from pomegranates, figs, and turnip greens, which are excellent sources of bioactive compounds [23,24,25,26]. These fruit and vegetables were selected as some of the most abundant agricultural productions of the Puglia region. As regards turnip greens, about 95% of the national cultivated area is in Puglia, Lazio, and Campania. The Puglia region is the second producer of pomegranate at the national level, after the Sicily region. The largest fig plantation in Europe is in Puglia, with 4500 fig trees divided into seven blocks of different varieties, all originating in Southern Italy and particularly in Puglia.

## 2. Materials and Methods

### 2.1. Raw Materials and By-Product Preparation

Pomegranates (*Punica granatum*, cv. Wonderful), figs (*Ficus carica* L.), and turnip greens (*Brassica rapa sylvestris*) were provided locally (Foggia, Italy). All the fruit and vegetables were washed with water, then dipped into chlorinated water (20 mL/L) for 5 min, and finally rinsed with sole water and air dried. Afterwards, the peels of pomegranates and figs were separated using a kitchen knife. For the turnip greens, the stems as by-products were separated from the tops and leaves. Then, all the by-products were dehydrated in a conventional dryer (PF–SIC-CO80PRO, SICCOTECH, Campobasso, Italy) at 60 °C and then milled into a fine powder with size ˂ 500 µm.

### 2.2. Shelled Shrimp-Based Burger Production

For the burger preparation, frozen shelled shrimps, potato flakes, potato starch, extra virgin olive oil, pepper, and spices were bought in a local market (Foggia, Italy). The shelled shrimps were defrosted under refrigerated conditions before burger preparation. Four types of shrimp-based burgers were prepared: a control sample (Ctrl) without any by-products (38.75 g shrimps, 3.87 g potato flakes, 2.42 g potato starch, 4.84 g extra virgin olive oil, 0.04 g pepper, and 0.08 g spices) and three samples containing the three by-products (2.91 g/by-product), corresponding to 7.5% (*w*/*w*) by-product in each burger. Percentages lower and higher than 7.5% were preliminary tested in our laboratory for the sensory acceptability of shrimp-based products (5%, 10%, 15%). The best sensory scores with each of the selected by-products were recorded with 7.5% addition (data not published). The fortified burgers were named as PP (pomegranate peel), TGB (turnip green by-product), and FP (fig peel), according to the by-product added to the formulation. To better mix the powder with the rest of the ingredients, the two powders from pomegranate peel and turnip green by-product were previously hydrated (8.56 g water fore PP and 11.67 g for TGB) before the addition to the mixture; and for the sole formulation containing FP, a further 0.08 g of salt was added to improve the product taste. For the homogeneous mixture, a bowl with a spiral hook was used for 5 min (Multichef, Ariete, Firenze, Italy). Using a mini-burger mold, burgers with a diameter of 50 mm and a thickness of 10 mm were prepared. Each sample was packaged, using a pad, in commercial high-barrier bag, and kept under refrigeration (4 ± 1 °C). All the samples were examined on day 0, 1, 3, 6, 8, 10, 13, 15, 17, and 20 to monitor microbiological quality, pH, and sensory properties. Total phenols, flavonoids, and antioxidant activity were also measured on the by-products and on the burgers just after their production.

### 2.3. Total Phenols, Flavonoids, and Antioxidant Activity

The chemicals used for the analyses were: Folin–Ciocalteu reagent, gallic acid monohydrate, methanol, hydrochloric acid, Trolox (6-hydroxy-2,5,7,8- tetramethylchroman-2-carboxylic acid), 2,2-azino-bis (3-ethylbenzothiazoline-6-sulfonic acid), diammonium salt (ABTS), potassium persulfate, sodium nitrite, sodium hydroxide solution, and quercetin, supplied from Sigma-Aldrich (Milan, Italy). Anhydrous sodium carbonate was supplied from Carlo Erba (Milan, Italy). All the reagents were of analytical grade.

Chemical analyses were carried out on the powders of the by-products and on the burgers, after the proper extraction process. The extraction was carried out as described by Cedola et al. [27] with some modifications. Specifically, the burgers were dried (BINDER GmbH, Tuttlingen, Germany) at 35 °C and then milled. In total, 20 mL of acidified methanol (80% MeOH in H_2_O acidified with 1% HCl) was mixed with 2 g of dry sample. The mixtures were shaken for 2 h at 3000 rpm (HS 260 BASIC, IKA, Staufen, Germany) and then centrifuged at 10,000 rpm (5804R, Eppendorf, Milan, Italy), at 5 °C for 15 min, to recover the supernatant. Three extractions were carried out for each sample. As described by Panza et al. [19], the total phenolic compounds (TPC) were determined by the Folin–Ciocalteu colorimetric method. TPC was expressed as mg of gallic acid/g of dry weight, according to a calibration curve (3.12–100 mg/L; R^2^ = 0.999). As described by Cedola et al. [27], the total flavonoids (TFC) were determined by the aluminum chloride colorimetric method. TFC was expressed as milligrams of quercetin equivalent (QE)/gram of dry weight (dw) according to a calibration curve (6.25–400 mg/L; R^2^ = 0.995). According to Re et al. [28], the antioxidant activity was assessed using the ABTS method and expressed as milligrams of Trolox equivalents/gram of dry weight (dw). A calibration curve was built using Trolox as a standard at concentrations between 12.5 and 500 mg/L (R^2^ = 0.990). Triplicate measurements were made for each sample.

### 2.4. Microbiological Analyses and pH Measurement

During the entire observation period, the burgers were monitored for microbial proliferation. With this aim, at each sampling time, 10 g of the burger was weighed in a sterile stomacher bag, peptone water (1:10) was used for sample dilution, and the sample was homogenized with a Stomacher LAB Blender 400 for 120 s (Pbi International, Milan, Italy). Then, the homogenized samples were plated in Petri dishes to enumerate yeasts and bacteria. Plate Count Agar (PCA, Oxoid Ltd., Basingstoke RG24 8PW, UK) incubated at 30 °C for 48 h and 5 °C for 10 days was used for mesophilic and psychrotrophic bacteria, respectively; Pseudomonas Agar Base (PAB, Oxoid), with an added cetrimide fucidin cephaloridine (CFC) selective supplement, incubated at 25 °C for 48 h was used for *Pseudomonas* spp.; Iron Agar (IA) supplemented with 5 g/L NaCl and incubated at 25 °C for 3 days was used for hydrogen-sulfide producing bacteria (HSPB); IA, supplemented with 10 g/L NaCl and incubated at 15 °C for 7 days was used for psychrotolerant and heat-labile aerobic bacteria (PHAB); Violet Red Bile Glucose Agar (VRBGA, Oxoid) incubated at 37 °C for 24 h was used for Enterobacteriaceae. For repetitiveness, all the microbiological tests were carried out two times, using two different samples. The results of microbiological analyses were expressed as log CFU/g. The following microbial thresholds were considered for data elaboration: 5 × 10^6^ CFU/g for total viable mesophilic and psychrotrophic bacteria, 1 × 10^6^ CFU/g for *Pseudomonas* spp. and Shewanella, 1 × 10^7^ CFU/g for *Photobacterium phosphoreum* [17]. According to previous studies carried out on fish shelf life, the microbiological acceptability limit (MAL) was calculated. This parameter was intended as the number of days to reach the microbiological threshold [17,19].

The pH was measured two times on two different samples, using the first sample dilution. With the aim, a pH meter (Crison, Barcelona, Spain) was used after proper calibration.

### 2.5. Sensory Analysis

All the shrimp-based burgers were evaluated using a quantitative descriptive analysis (QDA). To the aim, a panel of seven trained judges (females, aged between 28 and 48 years), selected among the researchers of the Food Department was adopted. The panel had a high level of experience in evaluating fresh fish products before the current study, and for this reason a sole session of 1 h was necessary to define the sensory attributes to be considered. They tested both raw and cooked burgers, after cooking at 200 °C for 15 min in an electric oven. The judges were asked to give their opinion on the samples using as attributes odor, color, appearance, texture, and overall acceptance (overall quality), in a proper selected scale. In the scale, 9 corresponded to excellent, 8 to very good, 7 to good, 6 to reasonable, 5 to the acceptable limit, 4 to dislike, 3 to bad, 2 to very bad, and 1 to completely unacceptable [29]. Considering that small samples of raw and cooked products were prepared using food-grade ingredients and according to good manufacturing practices, no associated risk for the panelists was found, and therefore no Ethics Committee approval was required. Before the sensory analysis, all the panelists were fully informed of the research being carried out and sensory data utilization. Data of the overall quality were fitted by a mathematical model also used in other studies [19,29] to quantify the parameter of the sensory acceptability limit (SAL), which was intended as the number of days to reach the sensory threshold (score = 5).

### 2.6. Statistical Analysis

A one-way ANOVA analysis was used to statistically compare experimental data and fitting parameters (MAL and SAL). To determine significant differences among samples, Duncan’s multiple range test, with the option of homogeneous groups (*p* < 0.05), was carried out. STATISTICA 7.1 for Windows (StatSoft, Inc., Tulsa, OK, USA) was adopted as the data analysis and visualization program.

## 3. Results and Discussions

### 3.1. Total Polyphenol, Total Flavonoids, and Antioxidant Activity of Shrimp-Based Burgers

Table 1 collects the experimental data of total polyphenols, total flavonoids, and antioxidant activity of the three selected by-products. In each column, it is possible to find a significant difference (*p* < 0.05) among samples, in great favor of the pomegranate peel. This by-product is very interesting compared to the other two. Our findings are not surprising because the literature evidence also confirms that pomegranate peel can be considered one of the richest by-products among many fruits and vegetables of the Mediterranean area. It is widely recognized that pomegranate peel, which accounts for 50% of the fruit’s weight, generally contains flavonoids, tannins, and phenolics with antimicrobial and antioxidant properties [30]. Also, the correlation between total phenols and antioxidant activity was in accordance with most articles found on the topic [20,31].

Table 2 collects the experimental data of the total polyphenols, total flavonoids, and antioxidant activity of the burgers with and without by-products. As can be seen, statistically significant differences (*p* < 0.05) were found between the control and enriched samples for all three parameters investigated, thus suggesting that the addition of by-products to the formulation can enhance the antioxidant potential, regardless of the type of vegetable residue. Comparing the three by-products, it is possible to infer that pomegranate peels are the most interesting, being able to promote a significant increase above all in total phenols and consequently also in the antioxidant activity of final products. These results are in line with those recorded on the three by-product powders because, although at a reduced level for dilution effects or interactions with other ingredients, the highest concentrations of bioactive compounds and the most interesting antioxidant activity were found with burgers fortified with pomegranate peel, thus also confirming the good composition of this residue when mixed into the food formulation [32,33,34].

### 3.2. Quality Preservation of Shrimp-Based Burgers

This research was aimed to verify whether the addition of by-products to the formulation based on shelled shrimps would promote a better preservation of its quality during storage. Therefore, burger quality was assessed during about three weeks under a refrigerated temperature. As regards the microbial proliferation, the data trend was similar in the various samples, even though some differences were underlined among them. For each microbial group, in the control the bacteria grew more rapidly than in the burgers with by-products. In the samples with PP, the delay in microbial proliferation was more marked because the antimicrobial activity of this peel is more pronounced [34]. To better estimate the differences among samples, the mathematical approach also reported in other studies was adopted. The MAL values, as the number of days to reach the threshold, were used to assess the effects of each by-product on the different microbial species and make a more quantitative comparison among the burgers. All of the MAL values calculated by the fitting procedure are reported in Table 3 and can be properly used to underline the difference among samples. As can be observed, for each spoilage group, the control lasted less time compared to the other fortified products. The total mesophilic and psychrotrophic bacteria seem to be the most rapid microorganisms to proliferate, up to and exceeding the threshold set at 5 × 10^6^ cfu/g. Regardless, also in these two cases the control became unacceptable after 7–8 days, whereas the other fortified foods remained acceptable for a longer time, thus demonstrating the effects of by-products in controlling microbial proliferation due to their well-known antimicrobial effects. Comparing the MAL values of the three fortified samples, slightly better effects on mesophilic and psychotropic bacteria were found with burgers enriched with pomegranate peels [34]. As regards Enterobacteriaceae, in Table 3, it is striking to observe that control burger was found acceptable for about 10 days; the fig peels promoted a longer preservation for another week, whereas both PP and TGB allowed maintenance of the cell load below the threshold (10^4^ cfu/g) for the entire observation period.

Therefore, in Table 3, an MAL higher than the monitoring time was reported for these two samples. In terms of the *Pseudomonas* spp., both the control and the burger with TGB lasted about 12 days, whereas the other two fortified burgers always remained acceptable (Table 3). Looking at Table 3, it is possible to see that *Shewanella* spp. rapidly grew in all of the samples, except in the product containing the peel from pomegranate. As regards *P. phosphoreum,* while the FP slightly increased the storability with respect to the control (*p* > 0.05), the other two by-products were effective in significantly inhibiting microbial growth. Considering all the MAL values recorded for the various spoilage microorganisms, despite some important effects of by-products on microbial growth, it is possible to sum up that the main microorganisms that controlled spoilage in these products were the psychrotrophic bacteria because in all the samples a high proliferation occurred. The control remained acceptable for one week and a slight increase in storability was recorded with the other three different fortified burgers, without any statistically significant differences among them (*p* > 0.05). Among the by-products, the peels from pomegranate were found to be the most effective against all the spoilage groups, thus confirming data from the literature about the antibacterial and antifungal activity of pomegranate peel powder [33,34]. The mechanisms by which the bioactive components of pomegranate peel exert their activity have not been completely elucidated, although a consistent quantity of information is now available suggesting both a direct antimicrobial activity and the activation of resistance responses in treated plant tissues [35]. Numerous studies have correlated the antifungal and antibacterial activity of pomegranate peel to polyphenols, particularly punicalagins that account for more than 90% of peel phenolics. Punicalagin is the main bioactive compound of ellagitannins that are phenolic compounds typical of pomegranate and a few other plant species [36]. Comparisons with other data from the scientific literature dealing with shrimps are not very viable because only very few studies were found on shrimp-based burgers or patties and most of them were focused on strategies to improve the technological quality and not on the quality maintenance during a proper refrigerated storage period [7,21].

**Figure 1:**
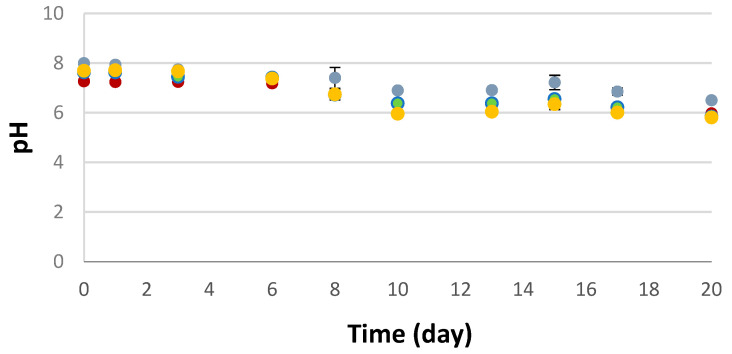
pH data in the control and in the fortified samples stored at 4 °C. Data are presented as the mean ± SD (*n* = 3). Grey: raw burger without powders; Red: raw burger with pomegranate peels; Green: raw burger with turnip green by-products; Yellow: raw burger with fig peels.

The values of pH are reported in Figure 1. As can be observed, the pH decreased in all of the samples. At the beginning of storage, the values ranged between 7 and 8, and after 10 days, up to the end of the observation period, they ranged between 6 and 7. This change during the storage time can be ascribed to microbial proliferation which became very high after 10 days of storage [17,18] and in part it might be attributed to the breakdown of glycogen with the formation of lactic acid [37]. The striking feature of the graph is that the control maintained slightly higher values than the other fortified samples, thus confirming that the addition of by-products can reduce the pH due to the presence of phenolic compounds [38,39]. No statistically significant differences (*p* < 0.05) among samples fortified with by-products can be highlighted, probably due to the low percentage of the by-product adopted in the formulation.

**Figure 2:**
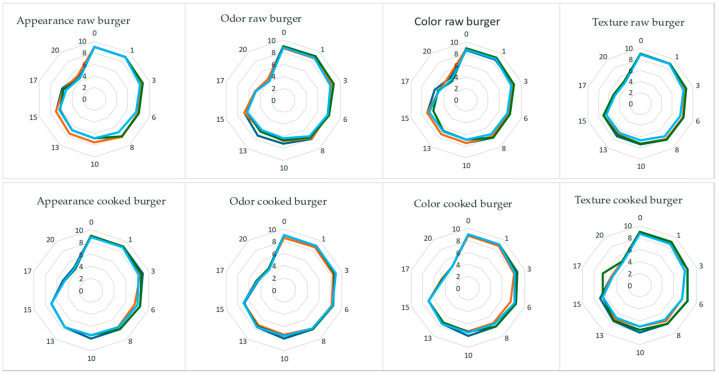
Radar map of sensory data of both raw and cooked control and fortified samples stored at 4 °C. Blue: raw burger without powders; Red: raw burger with pomegranate peels; Green: raw burger with turnip green by-products; Sky-blue: raw burger with fig peels.

As regards the sensory data, the results demonstrated that all of the shrimp-based burgers remained acceptable for less than 3 weeks, due to alteration of the main sensory attributes. Figure 2 reports graphs of each sensory parameter for both the raw and cooked products. As can be seen, in each graph the control and the various shrimp-based burgers can be compared. It is striking to observe that for two weeks all the products remained completely acceptable with score values higher than the threshold for acceptability (score around 7). Due to the proper burger sensory optimization carried out before the shelf life test, the by-product addition did not affect any sensory index. From the 17th day of storage, some changes occurred in all the samples and for all the sensory parameters, that became completely unacceptable between day 17 and 20. These sensory changes can be justified with the high proliferation of microorganisms that generally compromises product acceptance by provoking defects in appearance, odor, color, and texture [19,29]. As a fact, after 15 days of storage the burgers were highly contaminated and it was possible to assume the production of Trimethylamine, resulting from the bacterial reduction in Trimethylamine oxide. It is associated with the fishy odor of spoiling seafood [40]. The other important feature of this figure is the lack of any differences between the control and fortified burgers during time, thus demonstrating that the evolution of sensory properties after two weeks of storage did not depend on the by-product used in the formulation.

The adoption of the same equation used for microbiological data allowed calculation of the sensory acceptability limit (SAL), as the number of days within the product sensory scores remained below the threshold (score = 5). To this aim, data of the overall quality were fitted. Table 4 listed the results of the SAL values for each sample.

As can be seen, no differences appeared among the samples and between the raw and cooked products. All the samples lasted about 18 days, thus demonstrating that the addition of by-products to the formulation did not affect the product acceptability. Previous experimental findings recorded on the topic demonstrated the possibility to fortify fish-based food with fruit and vegetable by-products without compromising food acceptance. Cedola et al. [16] optimized the addition of 10% (*w*/*w*) dry olive paste flour coming from the *Coratina* cultivar in fish burgers by proper hydration/extraction of the bitter components with milk, before mixing the by-products with the other food ingredients. Panza et al. [19] used an olive pomace obtained by a modern decanter that recovers a wet pulp without kernels as the process by-product, to bread cod sticks and, later, the same authors published a study [20] dealing with the use of pomegranate peel in the breading of cod sticks. In both cases, these researchers demonstrated that the selected by-products did not compromise the quality of cod sticks, on the contrary, they helped in maintaining good sensory properties during storage. The research of Di Lucia et al. [18] also assessed that the addition of prickly pear (*Opuntia ficus-indica*) peel to food formulation did not provoke sensory defects. More recently, Panza et al. [29] studied the application of the juice, peel, and seeds of pomegranate for a fresh fish shelf life prolongation. In this case-study, the authors also assessed that the cod-based samples remained acceptable for about 3 weeks.

Combining the results recorded on microbiological quality and sensory properties (Table 3 and Table 4), it is possible to infer that product shelf life strictly depends on the contamination level of the food matrix; in fact, the proliferation of psychrotrophic bacteria occurred after about 1 week in the control burger and after 8.5–9 days in the other samples. This experimental evidence allows demonstrating that the addition of by-products, and in particular of peel powder from pomegranate, can increase the storability of fresh burgers based on shelled shrimps by at least two days, without affecting the product sensory quality.

## 4. Conclusions

In this research, different by-products were separately used in shrimp-based burgers to preserve the product quality during storage. With this aim, the samples were monitored for about 3 weeks under refrigerated conditions. Interesting results were found from the microbiological point of view because spoilage bacteria, and in particular total mesophilic and psychrotrophic bacteria, proliferated more rapidly in the control sample compared to the fortified ones. The burgers containing pomegranate peels remained acceptable for more time, thus confirming the antimicrobial and antioxidant properties of this by-product. From the sensory point of view, all of the samples remained acceptable for about 18 days, regardless of the addition of by-products to the formulation. As expected, the by-product inclusion enhanced the content in total phenols, total flavonoids, and antioxidant activity of this product, especially when peel powder from pomegranate was the new ingredient of formulation. To sum up, the current case-study demonstrated the effectiveness of by-products in preserving fresh shrimp-based burger shelf life. The interesting results recorded with pomegranate peels suggest focusing attention on this by-product to carry out further studies aimed to identify the optimal concentrations of powder to be added to the formulation to maximize the effects on the food without compromising its acceptance, thus promoting the potential of by-products at the industrial level as an efficient and more sustainable strategy for quality control during proper refrigerated storage.

## Figures and Tables

**Table 1:** Total phenols, total flavonoids, and antioxidant activity of by-products.

Samples	Total Phenols (mg GAE/g dw) ± SD	Total Flavonoids (mg QE/g dw) ± SD	Antioxidant Activity (mg Trolox/g dw) ± SD
PP	20.52 ± 1.55 ^a^	12.60 ± 2.40 ^a^	12.63 ± 0.4 ^a^
TGB	0.75 ± 0.32 ^b^	0.62 ± 0.06 ^b^	2.06 ± 0.4 ^b^
FP	1.01 ± 0.10 ^b^	1.00 ± 0.17 ^b^	4.06 ± 0.83 ^b^

Data are presented as the mean ± SD (*n* = 3). Data in each column with different superscript lowercase letters (a,b) show significant differences among samples (*p* < 0.05). GAE = gallic acid equivalents; QE = quercetin equivalent; PP: pomegranate peel; TGB: turnip green by-products; FP: fig peel.

**Table 2:** Total phenols, total flavonoids, and antioxidant activity of raw burgers.

Samples	Total Phenols (mg GAE/g dw) ± SD	Total Flavonoids (mg QE/g dw) ± SD	Antioxidant Activity (mg Trolox/g dw) ± SD
Ctrl	0.14 ± 0.28 ^a^	0.31 ± 0.08 ^a^	0.29 ± 0.06 ^a^
PP	2.67 ± 0.24 ^c^	1.62 ± 0.21 ^c^	12.63 ± 0.41 ^d^
TGB	0.75 ± 0.32 ^b^	0.62 ± 0.06 ^a,b^	2.06 ± 0.48 ^b^
FP	1.01 ± 0.10 ^b^	1.00 ± 0.17 ^b^	4.06 ± 0.83 ^c^

Data are presented as the mean ± SD (*n* = 3). Data in each column with different superscript lowercase letters (a–d) show significant differences between raw control and active samples of burgers (*p* < 0.05). GAE = gallic acid equivalents; QE = quercetin equivalent; Ctrl: raw burger without powders; PP: raw burger with pomegranate peels; TGB: raw burger with turnip green by-products; FP: raw burger with fig peels.

**Table 3:** MAL values (days) calculated for the samples stored at 4 °C.

Samples	Enterob.	Mes.	Pseud.	Psyc.	Shew.	Phot.
Ctrl	10.28 ± 0.5 ^a^	8.7 ± 0.6 ^a^	12.41 ± 0.9 ^a^	7.73 ± 0.5 ^a^	10.68 ± 0.7 ^a^	20.83 ± 8.9 ^a^
PP	>20	13.12 ± 8.3e + 5 ^a^	>20	9.18 ± 0.5 ^b^	>20	>20
TGB	>20	13.45 ± 0.8 ^a^	12.96 ± 1.1 ^a^	8.55 ± 0.9 ^a,b^	12.55 ± 0.6 ^a,b^	>20
FP	16.17 ± 3.2 ^b^	9.94 ± 0.8 ^a^	>20	8.51 ± 0.8 ^a,b^	12.66 ± 2.7 ^b^	23.39 ± 96.4 ^a^

Data are presented as the mean ± SD (*n* = 3). Data in each column with different superscript lowercase letters show significant differences between raw control and active samples of burgers (*p* < 0.05). Ctrl: raw burger without powders; PP: raw burger with pomegranate peels; TGB: raw burger with turnip green by-products; FP: raw burger with fig peels. Enterob. = Enterobacteria; Mes. = mesophilic bacteria; Pseud. = *Pseudomonas* spp.; Psyc. = psychrotrophic bacteria; Shew. = Shewanella; Phot. *= Photobacterium phosphoreum.*

**Table 4:** SAL values (days) for the overall quality calculated for the samples stored at 4 °C.

Samples	Cooked Samples	Raw Samples
Ctrl	18.19 ± 0.65 ^a^	18.68 ± 0.64 ^a^
PP	18.52 ± 0.85 ^a^	18.63 ± 0.59 ^a^
TGB	18.27 ± 0.73 ^a^	17.53 ± 0.69 ^a^
FP	18.28 ± 1.18 ^a^	17.25 ± 0.95 ^a^

Data are presented as the mean ± SD (*n* = 3). Data in each column with different superscript lowercase letters show significant differences between the raw control and active samples of burgers (*p* < 0.05). Ctrl: raw burger without powders; PP: raw burger with pomegranate peels; TGB: raw burger with turnip green by-products; FP: raw burger with fig peels.

## Data Availability

The original contributions presented in the study are included in the article, further inquiries can be directed to the corresponding author.
